# Effect of In Ovo Supplementation of Slab51 Probiotic Mixture, Associated with Marek’s Disease Vaccine, on Growth Performance, Intestinal Morphology and *Eimeria* spp. Infection in Broiler Chickens

**DOI:** 10.3390/ani14233435

**Published:** 2024-11-27

**Authors:** Lucia Biagini, Livio Galosi, Adolfo Maria Tambella, Alessandra Roncarati, Danilo De Bellis, Stefano Pesaro, Anna-Rita Attili, Sara Berardi, Giacomo Rossi

**Affiliations:** 1 School of Biosciences and Veterinary Medicine, University of Camerino, 62024 Matelica, Italy; lucia.biagini@unicam.it (L.B.); adolfomaria.tambella@unicam.it (A.M.T.); alessandra.roncarati@unicam.it (A.R.); danilo.debellis@unicam.it (D.D.B.); annarita.attili@unicam.it (A.-R.A.); sara.berardi@unicam.it (S.B.); giacomo.rossi@unicam.it (G.R.); 2 Department of Agricultural, Food, Environmental and Animal Sciences, University of Udine, 33100 Udine, Italy; stefano.pesaro@uniud.it

**Keywords:** in ovo inoculation, chicken, probiotics, Marek’s disease vaccine, intestinal morphology, coccidiosis

## Abstract

The interest for in ovo feeding has grown in the last decades mainly concerning probiotics, live microorganisms that can actively interact with the embryo. The aim of this study was to evaluate the effects of a multi-strain probiotic diluted in Marek’s disease vaccine (MDV) on zootechnical performances, intestinal morphology and *Eimeria* spp. infection. One hundred and twenty eggs of Ross 308 broiler chickens were incubated until 18 d, when 105 fertilised and vital eggs were randomly allocated into three groups. A control group (C) was inoculated with MDV; two treated groups (P1 and P2) were inoculated with MDV and different concentrations of probiotics: 1 × 10^5^ CFU/100 μL in P1 and 1 × 10^6^ CFU/100 μL in P2. After hatching, chickens were separated into three replicates (10/replicate). Zootechnical parameters were determined. At the end of the cycle (35 d), chickens were slaughtered, and the intestine was collected for morphological analysis from nine chickens per group (three/replicate). *Eimeria* spp. oocyst shedding was determined weekly, and parasitic lesions were analysed on the histological sample. In ovo treatment with probiotic did not influence hatching rate but significantly improved body weight and positively influenced intestinal morphometric data compared to C. Oocyst shedding in faeces resulted in an increase in C, with significant differences at sampling performed at 14, 21 and 28 d of age. These results suggest that the tested probiotic compound is safe for in ovo supplementation and effectively improves zootechnical performances and coccidian resistance.

## Simple Summary

In the last decades, several researchers investigated the feasibility of in ovo administration of different compounds for promoting health and productivity in poultry. The use of various substances is described in the literature, including vaccines, drugs, hormones, competitive exclusion cultures, pre-, pro- and postbiotics and nutritional supplements. Injected substances, volumes, dosages or concentrations vary according to bird type, egg size, time and site of injection, incubation system and regimen, with variable and sometimes contrasting results. Several reports indicated that probiotics may be effectively used to fight intestinal bacterial infections and promote growth in chickens. In ovo Marek’s disease vaccination is a practice widespread throughout the world, and the association of this vaccine with a probiotic mixture could be performed at the industrial scale, limiting the additional costs. The application of a probiotic mixture, associated with the Marek’s disease vaccine and inoculated in ovo, was tested to evaluate benefits to the poultry industry in a pilot scale.

## 1. Introduction

During incubation, the chicken embryo is completely dependent on the contents of the egg. A rapid change in its metabolism takes place in the last third of incubation, starting with the ingestion of amniotic fluid at around embryonic day 17 and the internalisation of the yolk in the abdominal cavity [1,2]. In the modern poultry industry, access to feed after hatching is usually delayed by 48–72 h, and during this period, the yolk represents the only source of nutrients to support gastrointestinal tract development, considering that at the end of incubation glycogen stores are almost completely depleted [3,4]. At the same time, the developing chicken embryo is able to produce an immune response to a pathogen prior to hatch; a feature that is widely exploited for modern large-scale poultry production with the routine administration of in ovo vaccination for multiple pathogens, including Marek’s Disease (MD) and Infectious Bursal Disease (IBD) [5]. Another fundamental change that occurs between hatching and housing is the microbial colonisation of the gastrointestinal tract [6]. In fact, unlike mammals or natural-hatched birds, which receive a natural microbial inoculation during parturition or contact within the nest, in poultry production, the interaction between chickens and the hen is completely excluded, and the main source of bacteria is the environment [7]. Vertical transmission of intestinal pathogens and commensal bacteria from hens to eggs and offspring has been demonstrated, identifying a number of core genera that may be vertically transmitted from the hen’s intestine to the chick, with a contribution of both the hen’s intestine and oviduct [8,9,10]. However, after hatching, chickens’ gut microbiota will be strongly influenced by the environmental microbes, and pathogens can easily be introduced in this process [11,12].

A balanced microbiota is essential to achieve one of the poultry industry’s objectives, which is to minimise the prevalence of infections while optimising productive performance [13]. Poultry meat production accounts for approximately one third of the world’s total meat production [14] and there is now strong evidence that the misuse of antibiotics, especially as growth promoters, has contributed to the emergence of multi-drug resistant bacteria in animals and humans, leading to a restriction in the use of antibiotics and the search for effective alternatives [15,16].

For all these reasons, in ovo feeding (IOF), the provision of nutrients to chicken embryo during the incubation, has gained increasing interest. This administration is usually performed in the amniotic fluid at a late incubation stage [17]. This represents an optimal delivery site because it is ingested by the chicken during hatching and comes into direct contact with enterocytes [18]. Various elements such as vitamins, carbohydrates, hormones, probiotic and prebiotics have been supplemented with IOF, not only to provide nutrients but also to promote correct chicken development [19,20]. Probiotics, defined as live microorganisms capable of positively modifying the health of the host [21], can actively interact with the gastrointestinal system and its microbiota, bringing multiple benefits [22,23,24,25]. Thus, the administration of probiotics in ovo during the late incubation stage allows an early interaction with the chicken, leading to a direct impact on the composition of microbiota [26,27], intestinal development and growth performances [28,29,30], immune system functionality [31,32,33] and resistance to infectious agents [28,31,34,35,36,37]. Among these, coccidiosis, caused by *Eimeria* spp., is considered one of the most important challenges in the poultry industry, as it causes intestinal damage with impaired digestive processes, loss of nutrient absorption capacity, dehydration and increased susceptibility to other infections [38]. The use of probiotics, both inoculated in ovo or administered in water, is a natural alternative to reduce the infection rate and *Eimeria* spp. oocysts shedding [39,40]. Most of the effects observed after probiotics administration are dependent on concentration, bacterial strain, age and site of administration [19,41]; therefore, each new probiotic compound needs to be tested to verify its safety and efficacy, especially concerning in ovo administration. The aim of this study is to evaluate the effects of two different concentrations of a multi-strain probiotic administered in ovo in the amniotic fluid on hatching rate, zootechnical performances, intestinal morphology and *Eimeria* spp. infection.

## 2. Materials and Methods

### 2.1. Probiotic and Vaccine Preparation

A commercial combined live, cell-associated Marek’s Disease vaccine (MDV, Nobilis^®^ Rismavac + CA126, MSD Animal Health S.r.l., Milano, Italy) was used for in ovo immunisation of chickens against Marek’s Disease. One ampoule of the vaccine (1000 doses) was diluted in 100 mL of sterile diluent.

The commercially available multi-strain probiotic Slab51 (Ormendes SA, Jouxtens-Mezery, Switzerland) was used. This product contains 200 billion units of lactic acid bacteria per 1.5 g of product, composed of the following strains: *Streptococcus thermophilus* DSM 32245, *Bifidobacterium lactis* DSM 32246, *Bifidobacterium lactis* DSM 32247, *Lactobacillus acidophilus* DSM 32241, *Lactobacillus helveticus* DSM32242, *Lactobacillus paracasei* DSM 32243, *Lactobacillus plantarum* DSM 32244 and *Lactobacillus brevis* DSM 27961. The product was properly diluted using the MDV diluent. The last dilution was performed directly into the reconstituted MDV. The treatments included the following: the control group (C), inoculated with 100 μL of MDV; the treated group (P1), inoculated with 100 μL of MDV and 1 × 10^5^ CFU/100 μL of probiotics and the second treated group (P2), inoculated with 100 μL of MDV and 1 × 10^6^ CFU/100 μL of probiotics. Concentration was assessed according to previous trials [37].

### 2.2. Birds and In Ovo Treatments

One hundred and twenty eggs of Ross 308 broiler chickens were obtained from a commercial hatchery (Avizoo-Euroagricola s.s., Longiano, FC, Italy) and incubated under standard conditions (37.5 °C and 54% RH) in a MG100/150 incubator (FIEM S.r.l., Como, Italy). Dead embryos or unfertilised eggs were excluded from incubation by candling. At day 18 of embryonic life, 105 fertilised and vital eggs were randomly allocated into three experimental groups (35 eggs/group) and subjected to the in ovo procedure. After checking the exact position of the air chamber, all eggs were sanitised at the blunt end with 70% ethanol. A pilot hole was made with an 18-gauge needle to pierce only the shell without entering the air chamber. The needle was disinfected with 70% ethanol between injections. Administration into the amnion was performed with a 25-gauge needle (2.5 cm long) fitted to a 1 mL syringe, using a new sterile needle and syringe for each egg. After injection, the hole was sealed with glue and all eggs were transferred to separate hatching baskets. At hatching, the number of live hatched or unhatched chickens was counted to calculate the percentage of hatchability for each group.

### 2.3. Animal Housing and Diets

After hatching, the 3 groups were separated into 3 replicates of 10 animals each. Chickens were housed in 3 adjacent sheds (12 m^2^ each), separated in 3 pens by nets, and a replicate of each group was placed randomly in every shed to avoid environmental interference in this study. All the animals received the same commercial feed (Cruciani, Montappone, MC, Italy) ad libitum throughout the trial (Table 1) and tap water. All the animals were weighted weekly with an electronic balance (mod. ACS-A9, My Scale, Foggia, Italy), while feed consumption was registered daily. From these data, body weight (BW), average weekly body weight gain (BWG), average weekly feed intake (FI) and the feed conversion ratio (FCR) were determined. More specifically, for each replicate, the average weekly weight gain was calculated as the cumulative weight gain of the animals within a replicate/number of animals per replicate; weekly feed intake was obtained as the total weekly consumption/number of animals per replicate and the feed conversion ratio was obtained as the total weekly feed consumption/total weight gain.

### 2.4. Sample Collection and Processing

At the end of the zootechnical cycle (35 days of age), chickens were slaughtered by electrical stunning and bleeding in an authorised slaughterhouse, and 9 chicken per group (3/replicate) were randomly selected for sampling. For each bird, ∼3 cm long portions of the different intestinal segments (duodenum, jejunum, ileum and caecum) were collected and 10% buffered-formalin fixed for 24 h. The samples were dehydrated through a graded series of alcohol, cleared with a ethanol/xylene solution, before embedding in paraffin wax. Serial sections (3 µm thick) were cut, stained with Haematoxylin-Eosin and mounted for the observation under the optical microscope (Leica DM2500, Wetzlar, Germany). Serum samples were obtained at slaughtering and frozen at −80 °C for serological analysis. Marek’s disease antibodies were assessed using a commercial ELISA test kit (ABBEXA, Cambridge, UK).

### 2.5. Histology and Morphometric Measurement

Haematoxylin–Eosin-stained sections of 5 well-oriented villi of the duodenum, jejunum, ileum and cecum from each sampled animal were measured considering villus height (VH), villus width (VW), crypt depth (CD) and lamina propria width (LPW), using an image processing and analysis system (Leica Imaging Systems Ltd., Cambridge, UK). VH was measured from the top of the villus to the top of the lamina propria [42]. CD was measured from the base upward to the region of transition between the crypt and villus [43]. VW was measured at the widest area of each villus, whereas the villus width–crypt depth ratio was determined as the ratio of VH to CD. Villus surface area (VSA) was calculated using the formula (2π)(VW/2)(VL) [44].

### 2.6. Eimeria Oocyst Shedding and Histological Lesions Score

Considering that the birds were raised in a commercial poultry farm, natural coccidian infection could occur. To assess *Eimeria* spp. oocyst shedding, weekly, starting from 7 days of age until the end of the trial, 10 fresh faecal samples were collected per pen, pooled and kept in separate airtight plastic bags. After homogenisation, samples were stored at 4 °C until assessed for oocyst count, which was determined using a McMaster counting chamber and stated as oocysts per gram of excreta, as previously described [45]. Histological lesions caused by *Eimeria* spp. were scored with a 0–3 scoring system in the duodenum, jejunum, ileum and caecum, considering both distribution and severity of infection, adapting a model previously described [46]. Briefly, a sum of the distribution (A) and severity (B) of protozoal infection was performed, where A represents the distribution of developmental stages of *Eimeria* spp. along the examined intestinal segment (0 = no parasites; 1 = parasites in one 10× field; 2 = parasites in two 10× fields and 3 = parasites in three 10× fields), and B represents the severity of the infection within the examined fields (0 = parasites in 0% of villi; 1 = parasites in <25% of villi; 2 = parasites in 25 to 50% of villi and 3 = parasites in >50% of villi). Then, the sum of the two parameters was divided by 2, to obtain a final total score 0–3.

In the same intestinal segments, enterocyte proliferation and presence of mixed inflammatory infiltrate in the lamina propria of examined villi was performed. A score from 0 to 3 points was assigned for the following parameters: 0 = no inflammation/proliferation; 1 = mild inflammation/proliferation; 2 = moderate inflammation/proliferation and 3 = severe inflammation/proliferation. The score was applied in three microscopic fields and the average value was calculated.

### 2.7. Statistical Analysis

Cardinal data were summarised using the arithmetic mean and the standard error of the mean; they were assessed for the assumption of normality of the data distribution using the Shapiro–Wilk test. All normally distributed cardinal data were analysed with one-way ANOVA (Analysis of Variance) and the Holm–Šidák post hoc test; otherwise, statistical analyses were performed with a non-parametric approach. Ordinal data were summarised using the median and range and compared between groups using the Kruskal–Wallis test and Dunn’s multiple comparison test. Differences with *p* values < 0.05 were considered statistically significant. All data were analysed using GraphPad Prism 10 statistical software for MacOS, version 10.1.1-270 (GraphPad Software Inc., San Diego, CA, USA).

## 3. Results

### 3.1. Marek’s Disease Virus Antibodies

All animals in the three groups were positive for the qualitative Marek’s disease antibody test.

### 3.2. Hatchability and Post Hatch Performance

No relevant differences were recorded in hatching rates between the three groups (94.2% for groups P2 and C; 97.1% for group P1). Table 2 reports the body weight of the chickens evaluated weekly. Starting from 7 days of age and up to the end of the cycle at 35 days, in ovo treatment with probiotic significantly improved body weight compared to C. No differences are observed regarding hatching weight.

Growth performances (BWG, FI and FCR) are listed in Table 3, with significant differences evident mainly until 21 days of age, for all the parameters.

### 3.3. Intestinal Morphometry

The results of morphometric analysis of the different intestinal tracts are shown in Table 4. Briefly, in the duodenum all the parameters showed no significant differences (*p* > 0.05). In the jejunum, the P2 group presented an increase in VW, CD and VSA compared both to C and P1 and a reduced VH/CD ratio compared with P1. In the ileum, probiotic treatment increased VH and reduced VW especially in P1, compared to both of the other groups (*p* < 0.01). Lastly, cecal CD and LPW of the C group are increased compared to both *p* groups (*p* < 0.01).

### 3.4. Oocyst Shedding and Histological Lesions Score

**Figure 1:**
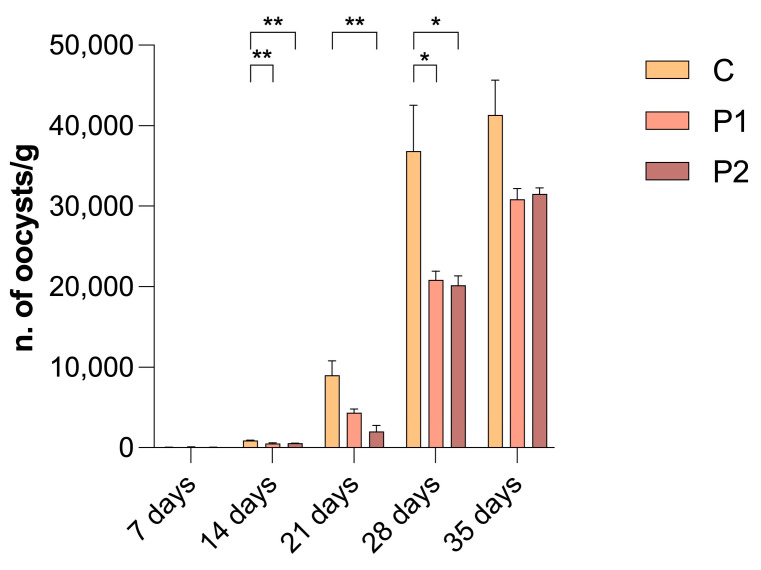
Mean number (and standard error) of coccidian oocysts per gram of faeces in the three groups; C: control group, MDV/100 μL; P1: MDV + 1 × 10^5^ CFU/100 μL of probiotic mixture; P2: MDV + 1 × 10^6^ CFU/100 μL of probiotic mixture. *p*-values, *: *p* < 0.05; **: *p* < 0.01.

Oocyst count in faeces showed a significant increase in C at sampling performed at 14, 21 and 28 days of age. No significant differences were observed at the end of the cycle (Figure 1).

**Figure 2:**
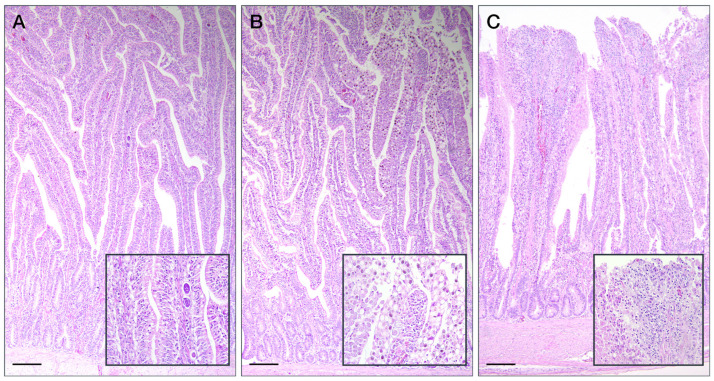
Coccidian infection in duodenum of broiler chickens. (**A**) In P1, mild infiltration of zygotes/macrogametes in the lamina propria was observed (insert), associated with the absence of lamina propria inflammation or enterocyte proliferation. (**B**) In P2, severe localised area of cellular swelling and hyperplasia (insert) of duodenal villous epithelium and lamina propria by zygotes/macrogametes was noted, associated with mild lamina propria inflammation. (**C**) In C, severe tissue damage was recorded, characterised by some areas of denudation of the villus apex due to apoptosis/rupture of the enterocytes (insert), associated with an obvious inflammation of the lamina propria, compatible with a strong proliferation of coccidia and the passage of zygotes/macrogametes in the faecal content immediately before slaughtering. Hematoxylin & Eosin, scale bar = 200 µm.

**Figure 3:**
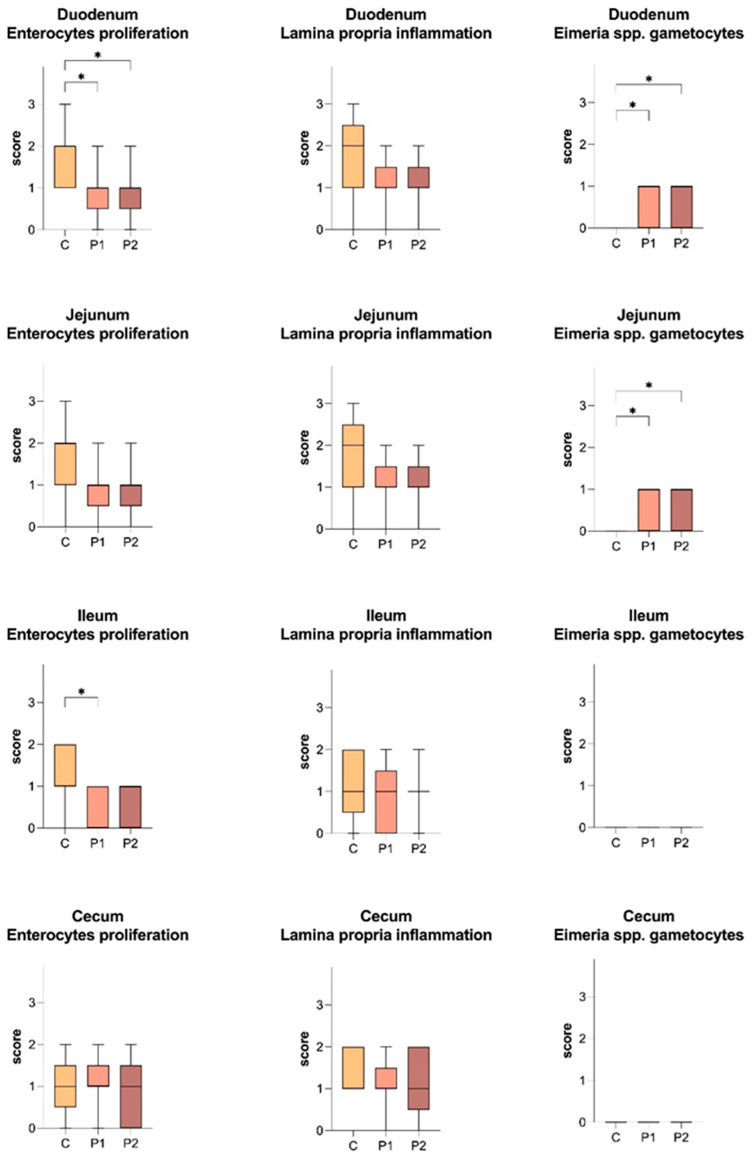
Boxplot showing the results of the scoring system detected in different segments of the intestinal tract (duodenum, jejunum, ileum and cecum) in chickens slaughtered at 35 days of age. C: control group, MDV/100 μL; P1: MDV + 1 × 10^5^ CFU/100 μL of probiotic mixture; P2: MDV + 1 × 10^6^ CFU/100 μL of probiotic mixture. The ends of the whiskers show minimum and maximum score values; boxes show the median, the first and the third quartile. Asterisks indicate significant differences between groups. *p*-values, *: *p* < 0.05.

At histological examination, the presence of *Eimeria* spp. was only revealed in the duodenum and jejunum of P1 and P2, with a median score of 1 (Figure 2 and Figure 3). Results of enterocyte proliferation and lamina propria inflammation are described in Figure 3.

## 4. Discussion

This study aimed to investigate the effect of the in ovo administration of two different concentrations of the probiotic mixture Slab51. This multi-strain probiotic has already been successfully tested as a feed supplement in avian species [47], but it is likely that not all probiotic bacteria commonly used as feed supplements are suitable also for in ovo injection. Therefore, a study was needed to evaluate the optimal concentration that could be safe for in ovo use and to assess the main effects of this administration over a 35-day life cycle.

In this trial, both the in ovo procedure and the probiotic supplementation in the amnios did not affect hatchability compared to the control group. As shown by previous works, no significant differences between controls and probiotic groups were found in the vast majority of studies reporting hatching rate [7,27,28,37,48,49,50,51,52], with a few reporting a negative hatching percentage [26,49]. These different results can be attributed to several influencing factors related to both the in ovo technique (volume of the injected solution, inoculation site, embryonic day of incubation and dilution vehicle) and to the probiotic characteristics (e.g., strain and concentration) [53]. For example, *Lactobacillus* spp.-based probiotics have already been proven to be safer for in ovo administration than those based on *Bacillus* spp. [49] due to a possible competition for nutrients of certain bacteria with developing embryonic cells. Most studies, including the present one, evaluated inoculation on incubation day 17 or 18, by which time most embryonic development has occurred, which allows both vaccination and in ovo feeding to be performed with the same injection, thus avoiding the increased risk of contamination due to the double injections necessary in the case of early administration (e.g., embryonic day 12). Concerning this trial, the combination of the probiotic with an MDV could represent another possible risk factor. Marek’s Disease is a lymphoproliferative disease of domestic chickens caused by an oncogenic α-herpesvirus with lymphotropic properties of gamma-herpesviruses, associated with lymphomas, neurological manifestations and immunosuppression [54], representing a major concern to the poultry industry. In ovo vaccination against this virus represents the only effective control method, due to the abundance and stability of the virus in the environment [55,56]. Previous studies showed that there is no negative effect on hatching rate after the combination of the vaccine with other compounds, such as probiotics. On the contrary, the early administration of the probiotic can promote the development of immunity [27,28]. Our results showed a favourable response to vaccination in all three groups suggesting both that there was no negative effect on MD vaccine efficacy following probiotic administration and that there was no direct stimulation of in ovo-administered probiotics in the production of Marek disease antibodies.

In terms of hatching weight, no significant differences between the different groups were observed in our work. Previous studies indicated that in ovo administration of *Bacillus subtilis* combined with *Bacillus amyloliquefaciens* [50], lactic acid bacteria [51], *Lactobacillus plantarum* + raffinose [26] and *Lactobacillus lactis* + *Bacillus subtilis* [57] resulted in an increased hatching weight, while others observed a reduction after the injection with *Lactobacillus animalis* + *Bifidobacterium animalis* or *Lactobacillus plantarum* + *Lactobacillus salivarius* [49,52]. However, the majority of the data reported are consistent with no significant results [7,27,28,48,49,58].

Among the after-hatching parameters, BW increased significantly in the two probiotic-injected groups throughout this study, with a gain of more than 100 g at 35 days of age compared with C. This stimulatory effect on body weight also emerges from a comprehensive review regarding the use of probiotics in ovo [53], and it is reported as an overall improvement in zootechnical performance (body weight, feed consumption and FCR) [26,28,57,58]. In fact, body weight data are often correlated with both feed consumption and intestinal morphology. For this specific probiotic mixture, dietary supplementation of Slab51 in Guinea fowls improved both BW and FCR [47]. Similarly, in this study, FCR is reduced in probiotic-treated chicken, with values of FI and FCR higher in the C group until 14 days of age, without statistically significant differences in the second part of the trial (14–35 days). All these data are consistent with the BW, indicating a better absorption capacity in the probiotic-treated groups. At the end of this study, P1 and P2 reached the higher mean weight with a lower total FCR, in line with other studies which applied in ovo probiotic supplementation [36,50,58,59].

Among the various factors influenced by the administration of probiotics that may explain the positive effects on animal performance are those related to intestinal morphology. The assessment of intestinal morphology is considered a reliable indicator of gut functionality, particularly in terms of the absorptive surface and subsequent transformation of nutrients [60], with studies describing both positive [47,61] or slight/absent [62,63] effects of oral probiotic supplementation on these parameters. With regard to in ovo administration, an improvement in gut morphology was observed in this study, which is consistent with previous data [27,28,35,48]. The variable extent of this improvement is attributable to sampling age, in relation to which a proportional increase occurs which makes it impossible to compare net data in relation to villus length or other parameters (e.g CD and VW). In our study, histomorphometric analysis confirmed that the use of probiotics induced an increase in villus height, with a gain of over 100 μm compared to group C, from the duodenum to ileum. Although not statistically significant, this difference observed at 35 days of age is a remarkable result given that it is achieved by a single administration during incubation and without any boost during the breeding period. In the jejunum, which is the main site of nutrient absorption, there was also an increase in VSA, further highlighting the improvement in the digestive capacity of the treated subjects. In addition, the HE analysis showed that the jejunum in the P1 and P2 groups exhibited intact histological structure, orderly arrangement, and well-grown intestinal villi with no obvious tissue damage and pathological changes, while the ileum in the C group appeared to be in the process of some structural alterations/lower VSA. Previously, Kim et al. (2012) [64] also reported that the administration of multi-microbe probiotic products increased the ratio of villus height to crypt depth in the jejunum. Consistently, this study provided evidence supporting the notion that supplementation with a multi-species probiotic mixture improves the intestinal structure by increasing the villus length. Other significant differences in the parameters evaluated for intestinal morphology were evidenced in this study, but without homogeneous results. It can be assumed that all these morphological changes are not only a direct effect of the interaction of probiotic bacteria with the intestinal mucosa, but also a result of the positive manipulation of intestinal microbiota by probiotic bacteria involved in the metabolism of nutrients as well as in the intestinal morphological development [65,66]. The gut microbiota constitutes the biological barrier that prevents pathogens from colonising the intestine and contributes to the maturation of the gastrointestinal tract and immune system [67]. The physical barrier of the intestinal epithelium, and especially the intestinal tight junction, confers the direct property of selective permeability to the gut [68]. In newly hatched chickens, the gastrointestinal tract is structurally and functionally immature, and maturation is induced by many factors, one of which is the presence and composition of gut microbiota. In both mammals and birds, studies have shown that the gut microbiota has a significant effect on gastrointestinal tract development: gut villus architecture, crypt depth, stem cell proliferation, blood vessel density, mucus layer properties and maturation of mucosal-associated lymphoid tissues are reduced in germ-free animals compared to those that are conventionally reared. Among the various stimulating factors produced by microbiota, in birds, enterocyte proliferation has been particularly linked to bacterial fermentation and the production of short-chain fatty acids (SCFAs), which are essential for enterocyte development and proliferation. [69,70,71,72]. Early intestinal colonisation with a positive microflora can, therefore, promote the proper maturation of the various intestinal components, including the related mucosal immune system, especially when this stimulation occurs before hatching in the final stage of incubation when organ development is almost complete.

Among the various factors that can negatively influences animal growth performance, there is *Eimeria* spp. infection. Many different probiotic compounds administered through the diet have been shown to provide a protective effect in chickens that were naturally infected or challenged with different *Eimeria* species [73,74,75,76]. Whereas, to date, the beneficial effects of probiotic bacteria administered in ovo to reduce the impact of coccidia on the intestinal mucosa have only been demonstrated in a limited number of studies in chickens and associated to exclusive probiotics effect [36,77] or to their combination with a coccidiosis vaccine [78]. In the present study, it was hypothesised that the beneficial effects of in ovo administration of a multi-strain probiotic mixture would lead to a stabilisation of the gut microflora by competitive exclusion of their pathogenic counterparts and, in addition, that the treatment would not lead to an increase in the severity of the disease induced by natural infection with *Eimeria* species, favouring, on the contrary, a reduction in clinical signs due to its beneficial effect on the development of the gut microbiota and immunity. One of the main mechanisms of action of probiotics is the challenge for receptor sites, which prevents the perforation and secretion of *Eimeria* sporozoites into the intestinal mucosa, resulting in reduced intestinal damage, proliferation and oocyst shedding [79]. Competition for receptors is even more limited in the case of IOF as the administration of the probiotic occurs before possible contact with the pathogen, which will, therefore, have limited receptor availability. In our study, from 14 days of age until the end of the trial, oocyst shedding was significantly reduced following in ovo probiotic supplementation, with no relevant differences among P1 and P2. The severity of the infection was assessed by histological evaluation of various parameters [80], and, in all the groups, the infection was limited to the upper gastrointestinal tract, without histological lesions in the ileum or caecum. The involvement of specific intestinal tracts depends on the specificity for the site of infection of the different *Eimeria* species that could be naturally present in the facility. Interestingly, in group C, which had the higher number of oocyst *per gram* of faeces, no macrogametes were histologically detected. This could be attributed to a different stage of the parasite life cycle, with oocysts being released from the ruptured epithelial cells and shed into the environment with the faeces at the time of organ sampling. In addition, although the protective mechanisms of probiotics are not fully understood, it has been shown that probiotics can significantly enhance mucosal-associated immune responses, increase the production of anti-Eimeria antibodies and reduce oocyst shedding [81]. Some studies have shown that the presence of IgA antibodies in the intestinal mucus, directed against some sporozoite-associated antigens, is able to modulate the degree of infection by coccidia and delay the release of oocysts [82]. In this case, it can be postulated that early intestinal colonisation with multi-species probiotic bacteria increased non-specific and specific mucosal defences, as well as the production of specific antibodies against protozoan antigens, leading to a delay in the sexual phase of the *Eimeria* species cycle and the release of oocysts, thus reducing the impact of coccidia on intestinal morphology. The results of the scores suggest a condition of severe inflammation of the lamina propria associated with enterocyte proliferation in the C group, especially in the duodenum, serving as further evidence of improved gut health and control of coccidian infection in probiotics-treated chickens. However, these histological changes could also be related to other causes of inflammation than just coccidian infection. In the caecum of group C, both the attributed scores and the histomorphometric measurements showed an increased enterocytic proliferation, CD, LPW and VW, suggesting a condition of widespread inflammation, with an increasing cellular turnover which is essential to replace the cellular damage.

The concentration of probiotics was chosen based on previous works that tested different compounds and concentrations for in ovo administration. In this study, no clear differences were observed on chicken’s performance or coccidian infection between P1 and P2; however, a slight reduction in hatchability percentage was observed in P2. Consequently, the concentration of 1 × 10^5^ CFU/100 μL could be more suitable for in ovo administration, even if further studies on an industrial scale are needed.

## 5. Conclusions

In conclusion, our data demonstrate that in ovo supplementation with Slab51 can be considered safe, and its association with MDV does not affect hatchability. Despite the lack of information on the microbiota composition of the animals included in this study, the efficacy of supplementation is demonstrated by the effects observed on productive performances, histological parameters and coccidian resistance, which were improved by the in ovo treatment.

## Figures and Tables

**Table 1:** Proximate composition of the feeds administered to the three groups during the trial.

	Feed I *Starter*	Feed II *Grower*
Age of broiler chickens (days)	1–15	16–35
*Proximate composition (%) wet weight basis*:		
Protein	22	21
Lipids	5	5.8
Ash	7.4	7.5
Fibre	4.4	4.5
Calcium	1	1.04
Phosphorum	1	0.85
Sodium	0.2	0.19
Lysine	1.2	1.2
Methionine	0.6	0.58
Phytasis (FTY)	1500	750
Endo-1.4-Beta-Xylanasis (FXU)	200	---
Vitamin A E672 (UI/kg)	12,000	4000
Vitamin D3 E671 (UI/kg)	2000	1250
Vitamin E (apha toc. 91%) (mg/kg)	40	20
Cupper E4 (mg/kg)	16	10
Selenium E8 (mg/kg)	0.16	0.2
Luthein E161b (g/kg)	---	41
Zeaxanthin E161 (g/kg)	---	8.4

**Table 2:** Effect of in ovo probiotic supplementation on body weight (g).

Days of Age	C	P1	P2	Statistical Data
0	46.32 ± 0.6196	45.84 ± 0.4692	46.10 ± 0.6080	F = 0.1756, *p* = 0.8391, p* = 1.0
7	104.5 ± 2.539 ^a^	156.6 ± 4.798 ^b^	147.0 ± 2905 ^b^	F = 60.74, *p* < 0.001, p* = 0.0006
14	297.2 ± 6.854 ^a^	353.0 ± 12.70 ^b^	326.2 ± 6.826	F = 9.159, *p* = 0.0002, p* = 0.0012
21	614.7 ± 13.50 ^a^	750.9 ± 12.82 ^b^	755.7 ± 16.82 ^b^	F = 22.41, *p* < 0.000, p* = 0.0006
28	1021 ± 26.79 ^a^	1223 ± 32.23 ^b^	1269 ± 26.19 ^b^	F = 20.97, *p* < 0.000, p* = 0.0006
35	1741 ± 31.1 ^a^	1839 ± 32.88 ^b^	1955 ± 32.84 ^c^	F = 10.98, *p* < 0.000, p* = 0.006

Data are reported as mean ± standard error of the mean. Different pairs of superscript letters on the same row indicate significant differences found at the post hoc multiple comparisons test. C: control group, MDV/100 μL; P1: MDV + 1 × 10^5^ CFU/100 μL of probiotic mixture; P2: MDV + 1 × 10^6^ CFU/100 μL of probiotic mixture. F: result of one-way ANOVA test between groups; *p*: *p*-value; p*: adjusted *p*-value with Bonferroni–Šidák correction.

**Table 3:** Effect of in ovo probiotic supplementation on body weight gain, feed intake and feed conversion ratios.

	C	P1	P2	Statistical Data
**Body Weight Gain (g)**				
Day of hatch to 7 days	58.22 ± 2.552 ^a^	110.8 ± 3.162 ^b^	100.9 ± 4.260 ^b^	F = 67.49, *p* < 0.0001, p* = 0.0005
7 to 14 days	192.7 ± 2.503	196.4 ± 0.5508 ^a^	179.3 ± 5.345 ^b^	F = 6.941, *p* = 0.0275, p* = 0.1301
14 to 21 days	317.4 ± 10.38 ^a^	397.9 ± 10.90 ^b^	429.4 ± 15.48 ^b^	F = 21.46, *p* = 0.0018, p* = 0.0090
21 to 28 days	404.2 ± 42.55	472.2 ± 22.96	513.0 ± 55.39	F = 1.678, *p* = 0.2637, p* = 0.7836
28 to 35 days	723.3 ± 100.6	616.1 ± 16.04	686.5 ± 21.52	F = 0.8228, *p* = 0.4833, p* = 0.9632
**Feed intake (g)**				
Day of hatch to 7 days	123.8 ± 1.618 ^b^	104.7 ± 6.819 ^a^	131.3 ± 2.179 ^b^	F = 10.47, *p* = 0.0111, p* = 0.0543
7 to 14 days	283.1 ± 6.823 ^a^	238.7 ± 1.953 ^b^	304.9 ± 0.7796 ^c^	F = 66.84, *p* < 0.0001, p* = 0.0005
14 to 21 days	370.5 ± 11.66 ^a^	488.3 ± 2.998 ^b^	508.1 ± 4.070 ^b^	F = 102.9, *p* < 0.0001, p* = 0.0005
21 to 28 days	816.3 ± 79.77	836.5 ± 1.770	830.5 ± 12.73	F = 0.0498, *p* = 0.9518, p* = 1.0
28 to 35 days	1111 ± 17.92	1121 ± 17.14	1062 ± 8.906	F = 4.340, *p* = 0.0683, p* = 0.2979
**Feed conversion ratio**				
Day of hatch to 7 days	2.136 ± 0.1189 ^a^	0.9495 ± 0.08403 ^b^	1.308 ± 0.07395 ^c^	F = 41.70, *p* = 0.0003, p* = 0.0018
7 to 14 days	1.469 ± 0.02469 ^a^	1.216 ± 0.01235 ^ac^	1.704 ± 0.05283 ^bc^	F = 50.32, *p* = 0.0002, p* = 0.0012
14 to 21 days	1.168 ± 0.02550	1.229 ± 0.02612	1.186 ± 0.03364	F = 1.197, *p* = 0.3651, p* = 0.9345
21 to 28 days	2.079 ± 0.3462	1.779 ± 0.07964	1.657 ± 0.1788	F = 0.8944, *p* = 0.4571, p* = 0.9744
28 to 35 days	1.608 ± 0.2569	1.822 ± 0.06035	1.550 ± 0.05723	F = 0.8442, *p* = 0.4753, p* = 0.9791
Day of hatch to 35 days	1.603 ± 0.1012	1.556 ± 0.0214	1.490 ± 0.05435	F = 0.7098, *p* = 0.5288, p* = 0.9891

Data are reported as the mean ± standard error of the mean. Different pairs of superscript letters on the same row indicate significant differences found at the post hoc multiple comparisons test. C: control group, MDV/100 μL; P1: MDV + 1 × 10^5^ CFU/100 μL of probiotic mixture; P2: MDV + 1 × 10^6^ CFU/100 μL of probiotic mixture. F: result of one-way ANOVA test between groups; *p*: *p*-value; p*: adjusted *p*-value with Bonferroni–Šidák correction.

**Table 4:** Morphometric measurement of different intestinal tracts (µm) at 35 days of age.

	C	P1	P2	Statistical Data
**DUODENUM**				
villus height (VH)	1349 ± 60.29	1448 ± 71.90	1469 ± 33.53	F = 1.238, *p* = 0.3078
villus width (VW)	117.3 ± 6.896	108.4 ± 5.112	100.3 ± 4.447	F = 2.339, *p* = 0.1180
crypt depth (CD)	196.7 ± 13.13	194.2 ± 11.00	223.7 ± 13.50	F = 1.695, *p* = 0.2048
lamina propria width (LPW)	209.7 ± 13.60	230.2 ± 17.58	256.8 ± 16.18	F = 2.210, *p* = 0.1316
villus surface area (VSA)	498.4 ± 39.41	493.3 ± 33.62	463.9 ± 25.54	F = 0.3119, *p* = 0.7350
VH/CD ratio	7.278 ± 0.7143	7.633 ± 0.4124	6.743 ± 0.2921	F = 0.7862, *p* = 0.4670
**JEJUNUM**				
villus height (VH)	946.3 ± 46.84	1035 ± 71.37	1139 ± 61.47	F = 2.521, *p* = 0.1014
villus width (VW)	115.3 ± 5.855 ^a^	121.1 ± 4.698 ^b^	126.6 ± 8.130 ^b^	F = 3.991, *p* = 0.0319
crypt depth (CD)	200.6 ± 9.506	186.7 ± 14.48 ^a^	250.3 ± 18.79 ^b^	F = 5.136, *p* = 0.0139
lamina propria width (LPW)	248.8 ± 13.38 ^a^	250.9 ± 20.71	322.1 ± 26.04 ^b^	F = 4.063, *p* = 0.0302
villus surface area (VSA)	339.6 ± 18.59 ^a^	397.5 ± 26.82 ^b^	449.9 ± 34.70 ^b^	F = 6.005, *p* = 0.0077
VH/CD ratio	4.870 ± 0.1904 ^b^	5.783 ± 0.2950 ^a^	4.762 ± 0.3480 ^b^	F = 3.865, *p* = 0.0351
**ILEUM**				
villus height (VH)	613.5 ± 27.26 ^b^	754.2 ± 33.76 ^a^	648.4 ± 25.60 ^b^	F = 6.347, *p* = 0.0061
villus width (VW)	130.7 ± 7.242 ^b^	101.2 ± 2.998 ^a^	132.5 ± 5.500 ^b^	F = 10.10, *p* = 0.0007
crypt depth (CD)	165.1 ± 8.229	160.3 ± 8.542	154.5 ± 6.815	F = 0.4551, *p* = 0.6398
lamina propria width (LPW)	208.9 ± 10.03	190.2 ± 13.36	181.3 ± 6.968	F = 1.823, *p* = 0.1832
villus surface area (VSA)	248.8 ± 11.01	240.6 ± 16.11	207.6 ± 16.68	F = 1.094, *p* = 0.3511
VH/CD ratio	3.869 ± 0.33	4.827 ± 0.2521	4.345 ± 0.2991	F = 2.628, *p* = 0.0929
**CECUM**				
villus height (VH)	338.2 ± 21.38	289.7 ± 14.00	344.6 ± 24.44	F = 2.159, *p* = 0.1373
villus width (VW)	149.5 ± 6.602	137.5 ± 9.463	169.3 ± 33.93	F = 0.6010, *p* = 0.5563
crypt depth (CD)	160.5 ± 15.07 ^a^	113.8 ± 2.500 ^b^	129.2 ± 6.305	F = 6.225, *p* = 0.0066
lamina propria width (LPW)	218.1 ± 13.26 ^a^	158.0 ± 6.118 ^b^	160.6 ± 10.53 ^b^	F = 10.67, *p* = 0.0005
villus surface area (VSA)	160.4 ± 15.27	126.2 ± 13.26	183.8 ± 40.34	F = 1.237, *p* = 0.3081
VH/CD ratio	2.245 ± 0.2089	2.566 ± 0.08593	2.707 ± 0.1237	F = 2.535, *p* = 0.1003

Data are reported as the mean ± standard error of the mean. The statistical significance was set at *p* < 0.05. Different pairs of superscript letters on the same row indicate significant differences found at the post hoc multiple comparisons test. C: control group, MDV/100 μL; P1: MDV + 1 × 10^5^ CFU/100 μL of probiotic mixture; P2: MDV + 1 × 10^6^ CFU/100 μL of probiotic mixture; *p*: *p*-value.

## Data Availability

All data are included in this manuscript.
